# Epidemiological Dynamics and Trends of Dengue Outbreaks in Sao Tome and Principe: A Comprehensive Retrospective Analysis (2022–2024)

**DOI:** 10.3390/tropicalmed10020034

**Published:** 2025-01-24

**Authors:** Sousa Lazaro, Vilfrido Santana Gil, Ivando Carvalho Viegas de Ceita, Isaulina Neto Viegas Barreto, Eula Carvalho Batista Sousa Maquengo, Andreza Batista de Sousa, Bakissy da Costa Pina, Tieble Traore, Alimuddin Zumla, John Otokoye Otshudiema

**Affiliations:** 1 Ministry of Health, Rua Patrice Lumumba, Sao Tome C.P. 23, Sao Tome and Principe; isabarreto95@gmail.com (I.N.V.B.); eulamaquengo@gmail.com (E.C.B.S.M.); andrezabatista864@yahoo.com.br (A.B.d.S.); costapina31@gmail.com (B.d.C.P.); 2 World Health Organization (WHO) Country Office, Country Preparedness & IHR (CPI) Department, Avenida Kwame N'Krumah, Sao Tome C.P. 287, Sao Tome and Principe; santanagilv@who.int; 3 Instituto Nacional de Estadística (INE), Largo das Alfândegas, Sao Tome C.P. 256, Sao Tome and Principe; ivando50@gmail.com; 4 World Health Organization (WHO), African Regional Office, Emergencies Preparedness and Response (EPR), Cité du Djoué, Brazzaville P.O. Box 06, Congo; traoret@who.int; 5 Division of Infection and Immunity, Centre for Clinical Microbiology, University College London, and NIHR Biomedical Research Centre, University College London Hospitals NHS Foundation Trust, London NW1 2PG, UK; a.i.zumla@gmail.com

**Keywords:** dengue, epidemiology, rainfall patterns, Sao Tome and Principe, public health interventions

## Abstract

Background: Dengue has emerged as a significant public health concern in Sao Tome and Principe, with the first documented outbreak occurring between 2022 and 2024. This study examined the epidemiological patterns, environmental determinants, and demographic characteristics of dengue transmission during this period. Methods: We conducted a comprehensive retrospective analysis of laboratory-confirmed dengue cases using national surveillance data, clinical records, and environmental monitoring data. Statistical analyses included demographic profiling, temporal trend assessment, and environmental correlation studies using multiple regression modeling. Results: Among 1264 laboratory-confirmed cases, we observed distinct age-specific vulnerability patterns, with the highest incidence rate in the 70–79 age group (829.6 per 100,000) despite most cases occurring in younger adults. Rainfall emerged as the strongest predictor of dengue transmission (r = 0.96, *p* < 0.001), explaining 92% of case variance in the regression model. Case distribution showed marked temporal variation, with 91.9% of cases reported in 2022, coinciding with exceptional rainfall (3205 mm). The overall case fatality rate was 0.71% (95% CI: 0.33–1.35), with significant quarterly variations. Geographical analysis revealed concentration in the Água Grande district (68.2% of cases). Conclusions: This first comprehensive analysis of dengue in Sao Tome and Principe demonstrates the crucial role of rainfall in disease transmission and reveals important age-specific vulnerability patterns. These findings provide an evidence base for developing targeted interventions, particularly during high-rainfall periods, and suggest the need for age-stratified clinical protocols in similar island settings.

## 1. Introduction

Dengue is one of the most significant mosquito-borne viral diseases globally, predominantly affecting tropical and subtropical regions where environmental conditions support vector proliferation and survival. The primary vectors, *Aedes aegypti* and *Aedes albopictus*, thrive in these regions, contributing to an estimated 390 million infections annually worldwide [1,2]. Recent systematic analyses have documented the expanding distribution of these vectors across Africa, with particular concerns for island nations where unique ecological conditions influence transmission patterns [3,4].

The epidemiology of dengue in African settings is characterized by complex vector ecology and environmental determinants. Entomological studies have demonstrated that both *Ae. aegypti* and *Ae. albopictus* have established populations across various ecological zones, significantly impacting transmission dynamics [5,6]. These vectors exhibit remarkable adaptability to diverse environmental conditions, particularly in urban and peri-urban settings where human activities create abundant breeding sites [7,8]. Recent research has highlighted the rapid geographical expansion of dengue vectors across Africa, influenced by climate change, urbanization, and human mobility patterns [9]. This expansion is especially concerning in island settings, where limited healthcare infrastructure and surveillance capabilities pose significant challenges for disease control [10].

The Sao Tome and Principe archipelago’s vulnerability to dengue transmission shares important parallels with other tropical island settings. Recent studies in Cape Verde, another African island nation, have documented similar challenges in vector control and disease surveillance, particularly in urban areas where *Ae. aegypti* breeding sites are abundant [11]. Like Sao Tome and Principe, Cape Verde has experienced recurring dengue outbreaks influenced by similar environmental and socio-demographic factors, including rapid urbanization and limited water infrastructure [11,12].

Environmental and socio-ecological patterns in São Tom Tomé and Príncipe reflect trends observed in other island settings. A systematic review published in July 2022 examining dengue outbreaks across six South West Indian Ocean (SWIO) islands (Comoros, Madagascar, Mauritius, Mayotte, Seychelles, and Reunion) revealed that while dengue virus circulation dates back to the early 1940s, recent years have shown marked transmission intensification. Since 2017, Reunion witnessed the cocirculation of three serotypes (DENV-1, DENV-2, and DENV-3) and an increased number of cases with severe forms and deaths, highlighting the critical need for enhanced surveillance and vector control strategies in island settings [13].

The first documented dengue outbreak in Sao Tome and Principe occurred in April 2022, when DENV-3 genotype III was identified as the causative agent, predominantly affecting the Água Grande district [14]. Although the Ministry of Health has implemented vector control measures and community education programs, specific resource and infrastructure limitations have hampered their effectiveness. Key constraints include limited laboratory diagnostic capacity, with dengue confirmation testing primarily concentrated at the Hospital Ayres de Menezes [14,15], challenges in vector surveillance and case detection systems common to the African context [16], and gaps in systematic case reporting due to limited diagnostic and surveillance infrastructure [14,15,16].

Despite substantial advances in dengue research, critical knowledge gaps persist in understanding transmission dynamics within island settings, particularly regarding temporal patterns and intervention effectiveness [9,10,11,12,13,14,15,16]. We aim to characterize the epidemiological patterns and determinants of dengue transmission during a major outbreak in Sao Tome and Principe between January 2022 and June 2024. Our primary objective is to identify key factors influencing dengue transmission through systematic analyses of temporal distribution patterns, demographic risk factors, and environmental determinants. Secondary objectives comprise the quantification of age-related mortality differences among laboratory-confirmed cases and the assessment of climatic variables’ influence on transmission dynamics. These research objectives address fundamental knowledge gaps while providing evidence for developing targeted control strategies applicable to similar island settings.

## 2. Materials and Methods

### 2.1. Study Design

We conducted a comprehensive retrospective observational study using a convergent parallel mixed-methods design to examine dengue outbreak patterns in Sao Tome and Principe from 2022 to 2024. The quantitative component encompassed analysis of clinical records, laboratory data, environmental surveillance, and intervention outcomes [17]. Through semi-structured interviews with healthcare providers, Ministry of Health dengue response managers, and key stakeholders, we gathered qualitative insights [18]. Data integration followed a rigorous three-phase analytical approach. We first developed a structured matrix framework that aligned quantitative metrics (epidemiological data, case numbers, and vector indices) with qualitative findings from stakeholder interviews and field observations. We then conducted parallel analyses of statistical trends and thematic patterns before employing convergent triangulation to systematically identify where these data sources confirmed, contradicted, or complemented each other. This methodological approach strengthened our understanding of intervention effectiveness and community response, particularly in high-transmission areas.

### 2.2. Geographical Study Area

**Figure 1:**
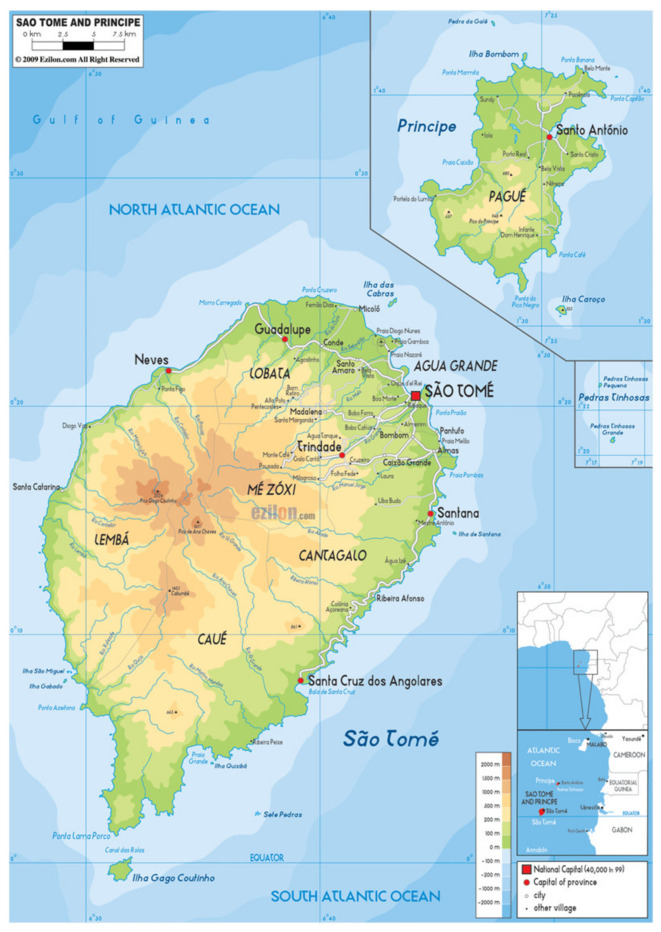
Map of Sao Tome and Principe (Source: Ezilon.com).

Sao Tome and Principe, a volcanic archipelago in the Gulf of Guinea (0.1864° N, 6.6131° E), encompasses two principal islands and adjacent islets off West-Central Africa (Figure 1). The mountainous landscape culminates at Pico de Sao Tome, rising to 2024 m. Characteristic of its equatorial position, the archipelago experiences a tropical rainforest climate where temperatures fluctuate between 23 and 30 °C throughout the year. The climate pattern follows a distinct rhythm, with prolonged rains from October through May, alternating with a drier period from June to September. Recent demographic data place the population at approximately 225,000 [19].

### 2.3. Study Population and Case Definition

Our research incorporated all dengue cases reported in Sao Tome and Principe during the study period. The case definitions were adapted from WHO guidelines to align with the Ministry of Health’s Standard Operating Procedures. The cases were classified into three categories. Laboratory-confirmed cases required positive results from either Real-time RT-PCR System (Applied Biosystems 7500, Thermo Fisher Scientific, Waltham, MA, USA) Dengue NS1 Ag STRIP (Bio-Rad Laboratories, Marnes-la-Coquette, France), or IgM/IgG serology/Dengue IgM/IgG Capture ELISA (Panbio, Abbott Laboratories, Abbott Park, IL, USA). Suspected cases presented with acute fever plus two or more characteristic symptoms (e.g., nausea/vomiting, rash, myalgia). Probable cases met clinical criteria with epidemiological links (contact within 14 days) to a laboratory-confirmed case. Cases presenting warning signs (severe abdominal pain, persistent vomiting, mucosal bleeding) or severe manifestations (shock, severe organ involvement) were prioritized for immediate hospital referral [20].

### 2.4. Data Collection and Sources

**Figure 2:**
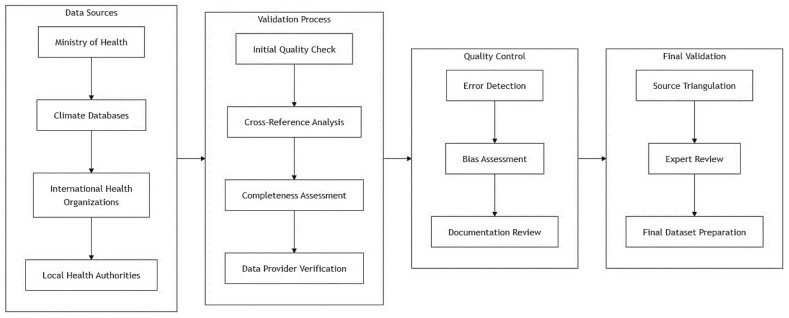
Flowchart of the data validation process.

Our data integration framework systematically consolidated information from multiple validated sources through a structured four-phase validation process (Figure 2). The primary data collection encompassed Ministry of Health surveillance records, hospital admission data, laboratory confirmation reports, and patient demographic profiles, with each dataset undergoing standardized quality assessment protocols. Environmental data, including daily temperature readings, rainfall measurements, and relative humidity recordings, were obtained from certified meteorological stations and integrated using automated data harmonization procedures.

To ensure data integrity and security, we implemented a comprehensive validation workflow beginning with initial quality assessment of individual datasets. This was followed by cross-reference analysis between different data sources to identify potential discrepancies or missing information. The completeness assessment phase evaluated data coverage across temporal and spatial dimensions, while data provider verification confirmed the authenticity and accuracy of source information. Quality control measures included systematic error detection, bias assessment, and thorough documentation review.

The final validation phase incorporated source triangulation and expert panel review, culminating in the preparation of the final integrated dataset. Throughout this process, we maintained strict adherence to data protection protocols, implementing encrypted data transfer systems and removing personal identifiers before analysis. All procedures received institutional ethical approval (Reference: STP/MOH/2024/03) and complied with national health data protection guidelines.

### 2.5. Data Analysis

Our statistical approach began with comprehensive assumption testing. Our analytical method integrated epidemiological indicators (calculated per 100,000 population) [21], environmental impact assessment through multiple linear regression [22], spatial analysis via QGIS (version 3.16.3, QGIS Development Team, Open Source Geospatial Foundation, Chicago, IL, USA) [23], and temporal trend examination using time-series methods [24]. The selection of climatic variables (temperature, rainfall, humidity) derived from established vector ecology literature [25] and local expert consultation. The multiple linear regression analysis verified normality through the Shapiro–Wilk test (*p* > 0.05), homoscedasticity via the Breusch–Pagan test, independence using the Durbin–Watson statistic, and multicollinearity through Variance Inflation Factors (VIF < 5). Highly correlated climate variables (r > 0.7) were combined into composite indices. The generalized additive models with cubic splines revealed significant non-linear relationships between climatic variables and dengue cases (*p* < 0.001). [26]

We calculated key epidemiological indicators using the following formulations:

Attack Rate = ((number of reported cases)/(total population)) × 100,000(1)

Incidence by Age Group = ((number of cases in age group)/(population of that age group)) × 100,000(2)

Proportion of Hospitalized Cases = ((number of hospitalized cases)/(total reported cases)) × 100,000(3)

Proportion of Severe Cases = ((number of severe cases)/(total reported cases)) × 100,000(4)

CFR = ((number of deaths due to dengue)/(total reported cases)) × 100(5)

Correlation and Regression Analysis: We examined associations between dengue cases and climatic variables using Pearson correlation coefficients. A multiple linear regression model was constructed to quantify the relationship between dengue cases (dependent variable) and four meteorological predictors: rainfall (mm), maximum temperature (°C), minimum temperature (°C), and wind speed (km/h). The model’s performance was assessed using R^2^, adjusted R^2^, F-statistic, and residual standard error. We calculated 95% confidence intervals for all regression coefficients, and statistical significance was set at *p* < 0.05. Model diagnostics included the assessment of residual normality, homoscedasticity, and independence.

Given the minimal missing data in our dataset (<5%), we employed complete case analysis after confirming, through Little’s MCAR test, that the data were missing completely at random. This approach maintained analytical integrity while avoiding the complexity and potential bias of imputation methods. We resolved data inconsistencies among sources through a hierarchical validation protocol, prioritizing laboratory-confirmed cases and implementing expert panel reviews for unresolved discrepancies. All analyses were performed using R (version 4.2.0, R Foundation for Statistical Computing, Vienna, Austria).

### 2.6. Ethical Considerations

This investigation received approval from the WHO AFRO Ethics Review Committee (AFR/ERC/2024/09.1) and the Sao Tome and Principe Ministry of Health Ethics Committee (STP/MOH/2024/03). Our protocol implemented comprehensive data protection measures, ensuring patient anonymity and secure data handling. Informed consent was waived for qualitative participants (health professionals), as no patients were directly involved. All research personnel operated under strict confidentiality agreements. The study adhered to the Declaration of Helsinki guidelines and local research ethics regulations.

## 3. Results

### 3.1. Demographic and Epidemiological Characteristics

Between January 2022 and June 2024, we documented 1264 laboratory-confirmed dengue cases in Sao Tome and Principe. Age-related mortality analysis revealed significant differences between fatal and non-fatal cases (Table 1). The median age of fatal cases was 44 years (IQR: 41–48) compared with 28 years (IQR: 15–41) in non-fatal cases (Mann–Whitney U = 3842, *p* = 0.038). Females comprised 63.6% of fatal cases versus 52.1% of non-fatal cases (χ^2^ = 8.24, df = 1, *p* = 0.004).

### 3.2. Age-Specific Incidence Rates

The highest age-specific incidence rates were observed in the elderly population (Table 2). The 70–79 age group showed the highest rate (829.6 per 100,000), followed by those aged ≥80 (800.0 per 100,000). Despite representing 25% of the population, the 0–9 age group showed the lowest incidence rate (280.9 per 100,000). The overall population incidence rate was 561.8 cases per 100,000 inhabitants.

### 3.3. Temporal Distribution

The epidemic showed substantial temporal variation (Table 3), with 91.9% (1161) of cases occurring in 2022, followed by a marked decline to 5.9% (75) in 2023 and 2.2% (28) in the first half of 2024. The highest disease burden was observed in the second quarter of 2022, with 863 cases (mean monthly cases 287.7 [SD 281.2]). Case fatality rates showed marked quarterly variation, peaking in the fourth quarter of 2022 (7.04%, 95% CI 2.33–15.67).

### 3.4. Climate Associations

Rainfall showed a strong positive correlation with dengue cases (r = 0.96, *p* < 0.001) and was the only significant predictor in the multiple regression model (Table 4). Temperature variables showed weak negative correlations with dengue cases, while wind speed showed negligible correlation. The regression model explained 92% of the variance in dengue cases (adjusted R² = 0.90, F = 57.57, *p* < 0.001).

## 4. Discussion

Our analysis of dengue cases from 2022 to 2024 reveals important patterns in demographics, geographical distribution, and environmental influences in Sao Tome and Principe. These findings provide crucial insights for public health interventions and outbreak management strategies.

Our findings contribute substantially to understanding dengue transmission dynamics in African island settings. The epidemiological patterns observed in Sao Tome and Principe align with recent research documenting the geographical expansion of dengue vectors across Africa—a phenomenon driven by climate change, urbanization, and human mobility patterns [9,10]. Particularly noteworthy are the parallels with Cape Verde, where similar challenges in vector control and disease surveillance have been documented, especially in urban areas with abundant Aedes aegypti breeding sites [11]. These similarities extend to the South-West Indian Ocean (SWIO) islands (Comoros, Madagascar, Mauritius, Mayotte, Seychelles, and Reunion), where a comprehensive systematic review published in July 2022 documented a marked increase in dengue transmission intensity. The review highlighted Reunion Island’s experience of continuous transmission since 2017, characterized by the co-circulation of three dengue virus serotypes (DENV-1, DENV-2, and DENV-3) [13]. This pattern of intensifying transmission across island settings underscores the critical importance of enhanced surveillance and vector control strategies, particularly in regions with limited healthcare infrastructure [10,11,12,13].

The age distribution analysis shows that while the 20–29 age group had the highest number of non-fatal cases, mortality was concentrated in the 40–49 age group. This pattern aligns with findings from recent studies in Southeast Asia [27], suggesting age-specific vulnerability patterns. The higher case numbers in young adults, coupled with lower mortality rates, may reflect both exposure patterns and age-related immune responses [28]. However, the increased mortality risk in middle-aged adults warrants particular attention in clinical management protocols.

The geographical analysis revealed a significant concentration of cases in the Água Grande district, accounting for 68.2% of all reported infections. This spatial clustering likely reflects the district’s urban characteristics, including population density and environmental conditions that favor vector breeding [29]. These findings emphasize the need for intensified vector surveillance and control measures in urban areas, alongside strengthened community engagement and healthcare capacity in affected regions.

The environmental analysis revealed complex relationships between climatic factors and dengue transmission. While dengue cases peaked during cooler months (May–July, 27.3–29.7 °C), this pattern coincided with peak rainfall periods (744–1186 mm), suggesting that rainfall may be a stronger driver of transmission than temperature alone [30]. The exceptionally high rainfall in 2022 (3205 mm) corresponded with the highest annual incidence rate (349.8 per 100,000), while reduced rainfall in subsequent years (159 mm in 2023, 16 mm in 2024) aligned with declining case numbers. Recent modeling studies in African island settings have shown that optimal temperatures ranging from 23 to 29 °C support dengue transmission through their effects on vector survival and viral replication [11,30]. While our study focused primarily on rainfall patterns, temperature data from Sao Tome and Principe’s meteorological records indicate relatively stable annual temperatures within this optimal range, suggesting that rainfall may be a more dynamic predictor of outbreak timing in this setting. Future studies combining long-term temperature and rainfall data would help quantify their relative contributions to dengue transmission dynamics.

Recovery profiles showed a mean duration of 6.81 days (SD: 6.84), indicating significant variability in clinical courses. This heterogeneity in recovery times emphasizes the need for individualized patient monitoring and flexible healthcare resource allocation [31]. The fluctuating case fatality rate across years (0.69% in 2022, 1.33% in 2023, and 0% in 2024) suggests improvements in case management but also highlights the need for sustained vigilance.

Different dengue serotypes can influence disease severity, outbreak dynamics, and population immunity patterns. Studies from similar island settings have shown that serotype shifts can drive outbreak intensity and clinical manifestations [11,15]. For instance, during Cape Verde’s 2009 outbreak, the identification of DENV-3 helped explain the observed clinical severity patterns and attack rates [11]. Given that this was the first characterized outbreak in Sao Tome and Principe, we limited our discussion to confirmed dengue cases without speculating about serotype-specific effects. Future surveillance incorporating serotype identification will be crucial for understanding local transmission dynamics and improving outbreak response strategies.

Several important limitations should be considered when interpreting our findings. The absence of serotype data due to laboratory capacity constraints represents a significant gap in our understanding of the outbreak dynamics. Without serotype identification, we cannot fully assess the relationship between specific viral strains and disease severity patterns. Furthermore, the lack of community-based seroprevalence studies limits our comprehension of the true infection burden, particularly regarding asymptomatic cases which often constitute a substantial proportion of dengue infections. The environmental data analysis was constrained by the relatively short study period, which may not capture longer-term climate patterns that could influence dengue transmission. The small number of fatal cases (*n* = 11) limits both the statistical power and the generalizability of our mortality analysis. Although we observed significant associations with both age and sex, sample size constraints preclude more complex multivariate analyses that could better elucidate the interplay among these factors. These findings should be considered preliminary and warrant further investigation in future studies with larger sample sizes. Additionally, our analysis of recovery times was based on available clinical records, which may not fully represent the spectrum of disease severity in the community [31].

Future research directions should prioritize the implementation of serotype surveillance systems and investigation of age-specific immune responses. Environmental modeling studies would enhance our ability to predict outbreak risks, while systematic evaluation of intervention effectiveness could guide resource allocation. The establishment of regular community-based seroprevalence surveys would provide valuable baseline data for future outbreak responses [32].

Our findings inform essential components of a comprehensive national dengue control strategy for Sao Tome and Principe. The significant spatial clustering of dengue cases in the Água Grande district (68.2% of total cases) demonstrates the necessity for targeted vector control interventions in this high-transmission zone. The robust temporal association between rainfall and dengue incidence (r = 0.96, *p* < 0.001) provides strong evidence for implementing season-specific vector control strategies, with our regression model explaining 92% of case variance (adjusted R² = 0.90, *p* < 0.001). The documented clinical manifestation patterns support the implementation of standardized case management protocols. These epidemiological findings indicate that vector control measures should prioritize the Água Grande district, while the integration of rainfall data into surveillance systems could strengthen outbreak prediction capabilities. Our results emphasize the need to enhance laboratory infrastructure for case confirmation and serotype surveillance, alongside the standardization of clinical management protocols based on local disease patterns. Systematic evaluation of these interventions, particularly in the Água Grande district, will be essential for evidence-based strategy adaptation. Community engagement programs should focus on this urban area where disease burden is highest, while health system strengthening should prioritize facilities serving affected populations.

## 5. Conclusions

This pioneering epidemiological analysis of dengue in Sao Tome and Principe establishes critical baseline data for understanding disease dynamics in small island settings. The clear association between rainfall patterns and disease transmission, coupled with distinct age-related vulnerability profiles, provides a robust foundation for evidence-based interventions.

The findings underscore the urgent need for three strategic improvements in dengue management. An integrated early warning system incorporating real-time rainfall data would enable proactive public health responses. Enhanced district-level vector surveillance, particularly in Água Grande, would strengthen targeted control efforts. Age-specific clinical protocols would address the observed disparities in disease outcomes across different population segments.

These insights extend beyond Sao Tome and Principe, offering valuable lessons for dengue management in resource-limited island settings. The documented relationship between environmental factors and disease patterns provides a framework for anticipating and responding to future outbreaks in similar geographical contexts. This understanding will prove particularly valuable as small island nations continue to face evolving public health challenges in the context of changing climate patterns.

## Figures and Tables

**Table 1:** Age-Related Mortality Analysis of Laboratory-Confirmed Dengue Cases, Sao Tome and Principe (2022–2024).

Age Parameters	Fatal Cases (*n* = 11)	Non-Fatal Cases (*n* = 1253)	Statistical Analysis
Median (IQR)	44 (41–48)	28 (15–41)	Mann–Whitney U = 3842
Mean (SD)	40.2 (17.9)	30.1 (18.2)	*p* = 0.038
95% CI of mean	28.2–52.2	29.0–31.2	
Age distribution test *	W = 0.892, *p* = 0.031	W = 0.967, *p* < 0.001	

* Shapiro–Wilk test for normality.

**Table 2:** Age-specific Dengue Incidence Rates in Sao Tome and Principe.

Age Group	Population *n* (%)	Cases (N)	Cases (%)	Incidence Rate Per 100,000
0–09	56,250 (25.0)	158	12.5	280.9
10–19	47,250 (21.0)	264	20.9	558.7
20–29	38,250 (17.0)	242	19.1	632.7
30–39	31,500 (14.0)	238	18.8	755.6
40–49	22,500 (10.0)	155	12.3	688.9
50–59	15,750 (7.0)	111	8.78	704.8
60–69	9000 (4.0)	59	4.67	655.6
70–79	3375 (1.5)	28	2.22	829.6
≥80	1125 (0.5)	9	0.712	800.0
Total	225,000 (100)	1264	100	561.8

**Table 3:** Temporal distribution of dengue cases and mortality in Sao Tome and Principe, 2022–2024.

Period	Cases	Deaths	CFR (%, 95% CI)	Mean Monthly Cases (SD)
2022				
Quarter 1	1	0	0 (0–97.5)	0.3 (0.6)
Quarter 2	863	1	0.12 (0.003–0.65)	287.7 (281.2)
Quarter 3	226	2	0.88 (0.11–3.15)	75.3 (48.1)
Quarter 4	71	5	7.04 (2.33–15.67)	23.7 (2.3)
Total	1161	8	0.69 (0.30–1.35)	96.8 (168.3)
2023				
Quarter 1	41	1	2.44 (0.06–12.85)	13.7 (8.1)
Quarter 2	21	0	0 (0–16.11)	7.0 (4.4)
Quarter 3	7	0	0 (0–40.96)	2.3 (1.5)
Quarter 4	6	0	0 (0–45.93)	2.0 (2.0)
Total	75	1	1.33 (0.03–7.21)	6.3 (6.1)
2024 *				
Quarter 1	15	0	0 (0–21.80)	5.0 (6.2)
Quarter 2	13	0	0 (0–24.71)	4.3 (4.0)
Total	28	0	0 (0–12.34)	7.0 (4.4)
Overall total	1264	9	0.71 (0.33–1.35)	

Data are *n*, %, or mean (SD). CFR = case fatality rate. * The data are available through June 2024 only.

**Table 4:** Relationship between climatic factors and dengue cases in Sao Tome and Principe.

Variable	Correlation *	Regression Coefficient ^†^ (95% CI)	*p* Value
Rainfall	0.96 (*p* < 0.001)	0.45 (0.38 to 0.51)	<0.001
Maximum temperature	−0.31 (*p* = 0.13)	2.21 (−18.84 to 23.26)	0.832
Minimum temperature	−0.23 (*p* = 0.26)	8.06 (−23.39 to 39.51)	0.608
Wind speed	0.02 (*p* = 0.93)	−1.15 (−17.43 to 15.13)	0.887
Model R^2^	0.92	-	<0.001

* Pearson correlation coefficients. ^†^ Multiple linear regression coefficients, adjusted for all variables.

## Data Availability

All of the relevant data generated during this study are included in the manuscript and its Supplementary Materials.

## Supplementary Materials

The following supporting information can be downloaded at: https://www.mdpi.com/article/10.3390/tropicalmed10020034/s1, Table S1. Descriptive Statistics of Dengue Case Age Distribution (2022–2024); Table S2. Distribution of Dengue Cases by District; Table S3. Monthly Overview of Dengue Cases and Climatic Factors (2022–2024); Table S4. Yearly Overview of Dengue Cases and Climatic Factors; Figure S1. Annual Incidence Rates of Dengue Cases by District in Säo Tomé and Principe, 2022–2024.

## References

[1] Bhatt S., Gething P.W., Brady O.J., Messina J.P., Farlow A.W., Moyes C.L., Drake J.M., Brownstein J.S., Hoen A.G., Sankoh O. (2013). The Global Distribution and Burden of Dengue. Nature.

[2] World Health Organization (2012). Global Strategy for Dengue Prevention and Control 2012–2020.

[3] Weetman D., Kamgang B., Badolo A., Moyes C.L., Shearer F.M., Coulibaly M., Pinto J., Lambrechts L., McCall P.J. (2018). Aedes Mosquitoes and Aedes-Borne Arboviruses in Africa: Current and Future Threats. Int. J. Environ. Res. Public Health.

[4] Kamgang B., Acântara J., Tedjou A., Keumeni C., Yougang A.P., Ancia A., Bigirimana F., Clarke S.E., Gil V., Wondji C.S. (2024). Entomological Surveys and Insecticide Susceptibility Profile of Aedes aegypti during the Dengue Outbreak in Sao Tome and Principe in 2022. PLoS Negl. Trop. Dis..

[5] Lutomiah J., Barrera R., Makio A., Mutisya J., Koka H., Owaka S., Koskei E., Nyunja A., Eyase F., Coldren R. (2016). Dengue Outbreak in Mombasa City, Kenya, 2013–2014: Entomologic Investigations. PLoS Negl. Trop. Dis..

[6] Zahouli J.B.Z., Koudou B.G., Müller P., Malone D., Tano Y., Utzinger J. (2017). Effect of Land-Use Changes on the Abundance, Distribution, and Host-Seeking Behavior of Aedes Arbovirus Vectors in Oil Palm-Dominated Landscapes, Southeastern Côte d’Ivoire. PLoS ONE.

[7] Ngoagouni C., Kamgang B., Nakouné E., Paupy C., Kazanji M. (2015). Invasion of Aedes albopictus (Diptera: Culicidae) into Central Africa: What Consequences for Emerging Diseases?. Parasit. Vectors.

[8] Kraemer M.U.G., Reiner R.C., Brady O.J., Messina J.P., Gilbert M., Pigott D.M., Yi D., Johnson K., Earl L., Marczak L.B. (2019). Past and Future Spread of the Arbovirus Vectors Aedes aegypti and Aedes albopictus. Nat. Microbiol..

[9] World Health Organization (2009). Dengue Guidelines for Diagnosis, Treatment, Prevention and Control: New Edition.

[10] Mavian C., Dulcey M., Munoz O., Salemi M., Vittor A.Y., Capua I. (2018). Islands as Hotspots for Emerging Mosquito-Borne Viruses: A One-Health Perspective. Viruses.

[11] Salgueiro P., Serrano C., Gomes B., Alves J., Sousa C.A., Abecasis A., Pinto J. (2019). Phylogeography and Invasion History of Aedes aegypti, the Dengue and Zika Mosquito Vector in Cape Verde Islands (West Africa). Evol. Appl..

[12] Yen T.Y., Cheng C.F., Tseng L.F., Carvalho R.M.C.A., Tsai K.H. (2024). Nationwide Inventory of Mosquitoes and the Distribution of Invasive Aedes (Stegomyia) albopictus (Skuse, 1894) on the Islands of Sao Tome and Principe in Central Africa. Insects.

[13] Hafsia S., Haramboure M., Wilkinson D.A., Baldet T., Yemadje-Menudier L., Vincent M., Tran A., Atyame C., Mavingui P. (2022). Overview of Dengue Outbreaks in the Southwestern Indian Ocean and Analysis of Factors Involved in the Shift Toward Endemicity in Reunion Island: A Systematic Review. PLoS Negl. Trop. Dis..

[14] World Health Organization Regional Office for Africa Disease Outbreak News: Dengue—Sao Tome and Principe. https://www.who.int/emergencies/disease-outbreak-news/item/2022-DON385.

[15] Kyeng M., Eric Y., Aliddeki D.M., Faria N.R., Kebede Y., Ndembi N. (2024). The Looming Threat of Dengue Fever: The Africa Context. Open Forum Infect. Dis..

[16] Zahouli J.B.Z., Utzinger J., Adja M.A., Müller P., Malone D., Tano Y., Koudou B.G. (2016). Oviposition Ecology and Species Composition of Aedes spp. and Aedes aegypti Dynamics in Variously Urbanized Settings in Arbovirus Foci in Southeastern Côte d’Ivoire. Parasit. Vectors.

[17] Martinez-Pulgarin D.F., Acevedo-Mendoza W.F., Cardona-Ospina J.A., Rodriguez-Morales A.J., Paniz-Mondolfi A.E. (2023). Epidemiological Research Methods in Infectious Diseases: Design and Analysis Considerations. BMC Infect. Dis..

[18] Santos Jose R.A., Moreira J., Peixoto T.M., Siqueira A.M., Lamas C.C. (2023). Mixed Methods in Public Health Research: Experiences from Dengue Studies. PLoS Negl. Trop. Dis..

[19] Instituto Nacional de Estatística São Tomé e Príncipe (2024). Population and Housing Census 2024.

[20] World Health Organization Regional Office for Africa (2024). Dengue in the WHO African Region: Situation Report 02 (14 January 2024).

[21] Li J., Liu X., Yang Y., Ma N., Guo Y., Gao J., Tang H., Xu K., Liu Q., Xu L. (2022). Climate Change Drives the Transmission and Spread of Vector-Borne Diseases: An Ecological Perspective. Biology.

[22] Kumar S., Srivastava A., Maity R. (2023). Modeling Climate Change Impacts on Vector-borne Disease Using Machine Learning Models: Case Study of Visceral leishmaniasis from Indian State of Bihar. Expert Syst. Appl..

[23] García-Suárez O., Tolsá-García M.J., Arana-Guardía R., Rodríguez-Valencia V.M., Talaga S., Pontifes P.A., Machaín-Williams C., Suzán G., Roiz D. (2024). Seasonal mosquito dynamics and environmental variables influence in Yucatan, Mexico. Acta Trop..

[24] Malik A., Yasar A., Tabinda A.B., Zaheer I.E., Malik K., Batool A., Mahfooz Y. (2017). Assessing spatio-temporal trend of vector breeding and dengue fever incidence in association with meteorological conditions. Environ. Monit. Assess..

[25] Palaniyandi M., Anand P.H., Manivel P., Thirumalai P., Sharmila T. (2021). Environmental determinants of dengue vector ecology: A space-time assessment using GIS and remote sensing applications. Environ. Sci. Pollut. Res..

[26] Rodriguez D.M., Rivera A., Sharp T.M., Ryff K.R., Pérez-Padilla J., Adams L.E., Rivera-Amill V., Paz-Bailey G. (2024). Epidemiology of Dengue—Puerto Rico, 2010–2024. MMWR Morb. Mortal. Wkly. Rep..

[27] Wilder-Smith A., Ooi E.E., Horstick O., Wills B. (2023). Dengue. Lancet.

[28] Wartel T.A., Prayitno A., Hadinegoro S.R., Medise B.E., Capeding M.R., Tam C.T., Bouckenooghe A. (2022). Three Decades of Dengue Surveillance in Five Highly Endemic South East Asian Countries. Vaccines.

[29] Vincenti-Gonzalez M.F., Grillet M.E., Velasco-Salas Z.I., Lizarazo E.F., Amarista M.A., Sierra G.M., Comach G., Tami A. (2023). Spatial Analysis of Dengue Seroprevalence and Modeling of Transmission Risk Factors in a Dengue Hyperendemic City of Venezuela. PLoS Negl. Trop. Dis..

[30] Wibawa B.S.S., Wang Y.C., Andhikaputra G., Lin Y.K., Hsieh L.H.C., Tsai K.H. (2024). The Impact of Climate Variability on Dengue Fever Risk in Central Java, Indonesia. Clim. Serv..

[31] Xu C., Xu J., Wang L. (2024). Long-term Effects of Climate Factors on Dengue Fever over a 40-year Period. BMC Public Health.

[32] World Health Organization Dengue in the WHO African Region: Situation Report 02: 14 January 2024. https://www.afro.who.int/countries/burkina-faso/publication/dengue-who-african-region-situation-report-02-14-january-2024.

