# Supplementary material for: Epidemiological Dynamics and Trends of Dengue Outbreaks in Sao Tome and Principe: A Comprehensive Retrospective Analysis (2022–2024)
Source: Trop Med Infect Dis. 2025 Jan 24;10(2):34. doi: 10.3390/tropicalmed10020034 (PMC11860262; doi:10.3390/tropicalmed10020034)
Supplement: Supplementary file 1 [file tropicalmed-10-00034-s001.zip › tropicalmed-3386077-supplementary.pdf]

Supplementary Materials

Table S1. Descriptive Statistics of Dengue Case Age Distribution (2022-2024)

Parameter	Value
**Mean**	30.2 years
**Median [95% CI]**	28 years [15–41]
**Mode**	15 years
**Standard Deviation**	18.2 years

Table S2. Distribution of Dengue Cases by District

District	Cases (N)	Percentage (%)
**Água Grande**	862	68.2
**Mézochi**	187	14.8
**Lobata**	106	8.39
**Cantagalo**	50	3.96
**Caué**	23	1.82
**Lembá**	22	1.74
**Pagué**	14	1.11

Table S3. Monthly Overview of Dengue Cases and Climatic Factors (2022-2024)

Month	Cases	Max Temp (°C)	Min Temp (°C)	Rainfall (mm)	Wind (km/h)
**January**	23	30.0	25.0	46	10.0
**February**	8	30.8	25.0	8	10.0
**March**	14	30.9	24.5	18	11.0
**April**	53	30.7	24.8	91	11.2
**May**	258	29.7	25.0	744	11.1
**June**	598	28.1	24.0	1186	12.0
**July**	123	27.3	24.0	244	13.0

**August**	80	28.0	24.0	314	14.0
**September**	30	28.4	24.1	336	13.3
**October**	25	28.8	24.0	175	11.0
**November**	27	29.6	24.1	185	11.0
**December**	25	30.3	24.2	33	10.2

Table S4. Yearly Overview of Dengue Cases and Climatic Factors

Year	Cases (N)	%	Max Temp (°C)	Min Temp (°C)	Rainfall (mm)	Wind (km/h)
**2022**	1161	91.9	29.8	24.2	3205	11.9
**2023**	75	5.93	30.2	24.7	159	11.1
**2024***	28	2.22	31.0	25.5	16	12.0

*Data available through June 2024 only

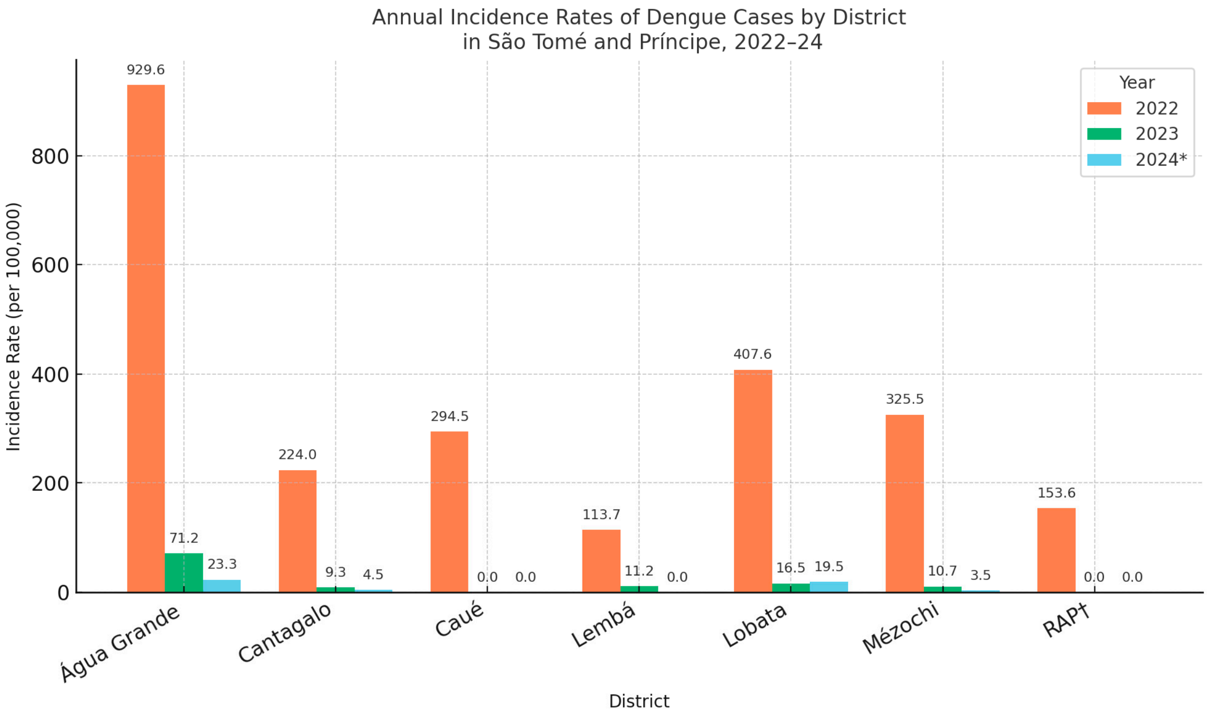

*Data available through June, 2024 only.

Figure S1. Annual Incidence Rates of Dengue Cases by District in São Tomé and Principe, 2022-2024
